# Standardized In‐house Protocol for Determining the Minimum Inhibitory Concentration (MIC) via Broth Microdilution for Natural Products Against Obligate Anaerobic Bacteria

**DOI:** 10.1002/cpz1.70474

**Published:** 2026-09-25

**Authors:** Daniela Silva Gonçalves, Mariana Brentini Santiago, Lucas Andrade Sousa, Carlos Henrique Gomes Martins

**Affiliations:** ^1^ Laboratory of Antimicrobial Testing, Institute of Biomedical Sciences Federal University of Uberlândia Uberlândia Minas Gerais Brazil; ^2^ These authors contributed equally to this work

**Keywords:** anaerobic bacteria, broth microdilution, in‐house protocol, minimum inhibitory concentration

## Abstract

Obligate anaerobic bacteria are important constituents of the human microbiota and are frequently involved in opportunistic infections. However, due to their fastidious nature and oxygen sensitivity, antimicrobial susceptibility testing (AST) is often neglected, resulting in empirical treatments and an increased risk of therapeutic failure. The broth microdilution method offers a cost‐effective and scalable alternative for antibacterial activity evaluation but is typically restricted to certain genera due to concerns over oxygen exposure. In this study, we present a complete standardized in‐house protocol for determining the minimum inhibitory concentration (MIC) of natural and synthetic antimicrobial agents against obligate anaerobes using the broth microdilution technique under strictly anoxic conditions. Our method is optimized for implementation in laboratories equipped with anaerobic chambers and allows high‐throughput screening using minimal sample volumes, making it ideal for natural product–based antibacterial discovery. The protocol is based on other guidelines with critical adaptations that ensure bacterial viability throughout the procedure. It provides reproducible results without oxygen interference, thus representing a valuable tool for researchers investigating novel antimicrobials targeting anaerobic pathogens, within a period of approximately 6 days. © 2026 The Author(s). *Current Protocols* published by Wiley Periodicals LLC.

**Basic Protocol 1**: Preparation of working solutions

**Basic Protocol 2**: Preparation of the 96‐well microplate

**Basic Protocol 3**: Inoculum standardization

**Basic Protocol 4**: Reading and interpreting the results

## INTRODUCTION

Obligate anaerobic bacteria are of great importance for the human microbiota, constitute mucosal surfaces such as the oral cavity and vaginal tract, and predominate the intestinal community by up to 93.31% (Gajdacs & Urban, 2020; Liu et al., 2024). During disruption of the mucosa or dysbiosis, anaerobic bacteria can also be considered opportunistic pathogens, causing infections either alone (monomicrobial) or with aerobic bacteria (polymicrobial) (Gajdacs & Urban, 2020).

Owing to the long generation time and rigorous growth conditions of these fastidious bacteria, isolation, identification, culture, and antimicrobial susceptibility testing (AST) are rarely performed in diagnostic or research laboratories. Consequently, anaerobic infections are historically treated empirically, and clinical failures lead to the evolution of antibiotic resistance, which results in a greater need for accessible antibacterial activity tests and new antibacterial agents (Dubreuil, 2024).

Over the years, natural products and their derivatives have been an abundant source of new therapeutic agents for many diseases, including antibacterial drug discovery (Xu et al., 2022). Newman and Cragg's review showed that from 01/1981 to 09/2019, of 1881 new approved drugs, 356 are derived from natural products, 71 are natural products, and 14 natural products are “botanical”; however, in relation to antibacterial drugs specifically, of 162 agents approved, 78 are derived from natural products, and 11 are natural products (Newman & Cragg, 2020).

However, conventional natural product extraction techniques require time, solvents, energy, and raw materials on a large scale for low yields (Ponphaiboon et al., 2023). One strategy to overcome this problem is the chemical synthesis of compounds found in nature. The total synthesis of active natural products solves the problem of limited natural resources, helps prepare a large number of active compounds, and enables the rapid synthesis of analogues with diverse skeletons and functional groups, building a natural product–like compound library to conduct extensive biological activity screening and drug development research (Hou et al., 2023).

According to the Clinical and Laboratory Standards Institute (CLSI) for anaerobic bacteria, the gold‐standard agar dilution method is ideal for surveillance and clinical decision‐making about the appropriate antimicrobial therapy for patient care, while the risk of prolonged exposure to oxygen during setup procedures limits the broth microdilution method to *Bacteroides* and *Parabacteroides* species (CLSI, 2018). The European Committee on Antimicrobial Susceptibility Testing (EUCAST) also recommends the agar dilution or disk diffusion method for anaerobic bacteria (EUCAST, 2025). Although EUCAST provides a rapid disk diffusion methodology validated for 16–20 h incubation, it can be used only for *Bacteroides* spp., *Prevotella* spp., *Fusobacterium necrophorum*, *Clostridium perfringens*, and *Cutibacterium acnes* (EUCAST, 2023).

Nevertheless, it has already been demonstrated that laboratories possessing anaerobic chambers are also able to use the broth microdilution method for testing more obligate anaerobic bacterial strains, but the diversity of methodologies is not adequately described. Not following recommended guidelines leads to considerable variability in results and makes studies difficult to compare (Goldstein et al., 2008; Reissier et al., 2023).

The agar dilution method is preferable for testing a large number of isolates with few dilutions of an antimicrobial agent. However, it is uneconomic, labor‐intensive, and inefficient for testing a single isolate or a small number of isolates, and the result is visual, requiring considerable expertise in setup and interpretation (Sood et al., 2022). The disk diffusion method offers a qualitative analysis, effective for rapidly multiplying bacteria, but not for obligate anaerobic bacteria; it has drawbacks, including low agar diffusion of some antimicrobial agents, which can lead to false‐negative results and difficulty in detecting resistance mechanisms (Elbehiry et al., 2025). In contrast, the broth microdilution method is preferable for testing a greater number of antimicrobial agents with more dilutions/concentrations at once, besides using a small volume of broth instead of macro agar plates, saving time and work by freezing the 96‐well plates already prepared with broth, and the result can be visual or quantified by photometry (Sood et al., 2022).

The standardized in‐house protocol presented in this work can be applied to determine the minimum inhibitory concentration (MIC) of natural products, their derivatives, isolated substances, and other synthetic compounds against obligate anaerobic bacteria via the broth microdilution technique. This protocol expresses consistent accuracy, good reproducibility, and a better cost–benefit relationship compared to agar dilution or disk diffusion methods. So far, it has been applied to determine the antibacterial activity of natural products such as *Copaifera pubiflora* oleoresin and its isolated compounds (Moraes et al., 2020); Brazilian red propolis crude hydroalcoholic extract, fractions, and isolated compounds (Silva et al., 2022); cannabidiol (Santos et al., 2025); and synthetic chalcones (Satokata et al., 2022) against periodontopathogenic bacteria such as *Porphyromonas gingivalis*, *P. asaccharolytica*, *Prevotella nigrescens*, *P. intermedia*, *P. oralis*, *P. buccae*, *Fusobacterium nucleatum*, *Actinomyces viscosus*, *A. naeslundii*, *Peptostreptococcus anaerobius*, *Parvimonas micra*, and *Veillonella parvula*. The experimental procedures provided here as a model have been applied mainly to periodontal pathogens but are encouraged to be used with other anaerobic bacterial species.

The main benefit of this present protocol is to ensure that AST for obligate anaerobic bacteria is performed more frequently and effectively, extending the advantages of the broth microdilution method to more bacterial genera, encouraging research into natural products, their derivatives, isolated substances, and other synthetic compounds as new antimicrobial agents. However, the major limitation of this protocol is the need for an anaerobic chamber in the laboratory. The anaerobic chamber, with its controlled atmosphere consisting of 10% CO_2_, 5%–10% H_2_, and 85% N_2_ at 37°C, establishes and maintains an ideal strictly anoxic environment for obligate anaerobic bacteria. These conditions ensure bacterial viability and growth for execution of the technique. Thus, all steps of the experiment involving manipulation of the bacteria take place inside the anaerobic chamber.

Our study group has been carrying out trials in search of new antimicrobials with this proposed technique for some years, publishing the methodology in a summarized manner. Now, with the complete protocol, we expect to help future researchers interested in the subject. The key stages of the broth microdilution method involve the preparation of working solutions (Basic Protocol 1), preparation of the 96‐well microplate (Basic Protocol 2), inoculum standardization (Basic Protocol 3), and reading and interpreting the results (Basic Protocol 4).

*CAUTION: P. gingivals* and *Bacteroides fragilis* are Biosafety Level 2 (BSL‐2) pathogens. These organisms should be handled using standard BSL‐2 practices and guidelines such as, but not limited to, wearing a laboratory coat, use of gloves, minimizing the use of sharps, and disposal of waste as a biohazard. All blood products must be handled as potentially biohazardous materials. Although sourced from healthy animals, strict adherence to standard biosafety precautions (specifically BSL‐2 practices) is mandatory when handling biological fluids to preclude accidental contamination. Metronidazole, sodium hydroxide, menadione, and dimethyl sulfoxide are hazardous. Standard personal protective equipment (PPE) must be worn, and these reagents should be handled inside a fume hood. All procedures in this article, unless otherwise indicated, should be completed using aseptic techniques, and all solutions and instruments that come into contact with living organisms should be sterile.

*NOTE*: All protocols involving animals must be reviewed and approved by the appropriate Animal Care and Use Committee and must follow regulations for the care and use of laboratory animals. Appropriate informed consent is necessary for obtaining and use of human study material.

## Basic Protocol 1 PREPARATION OF WORKING SOLUTIONS

**Figure 1:**
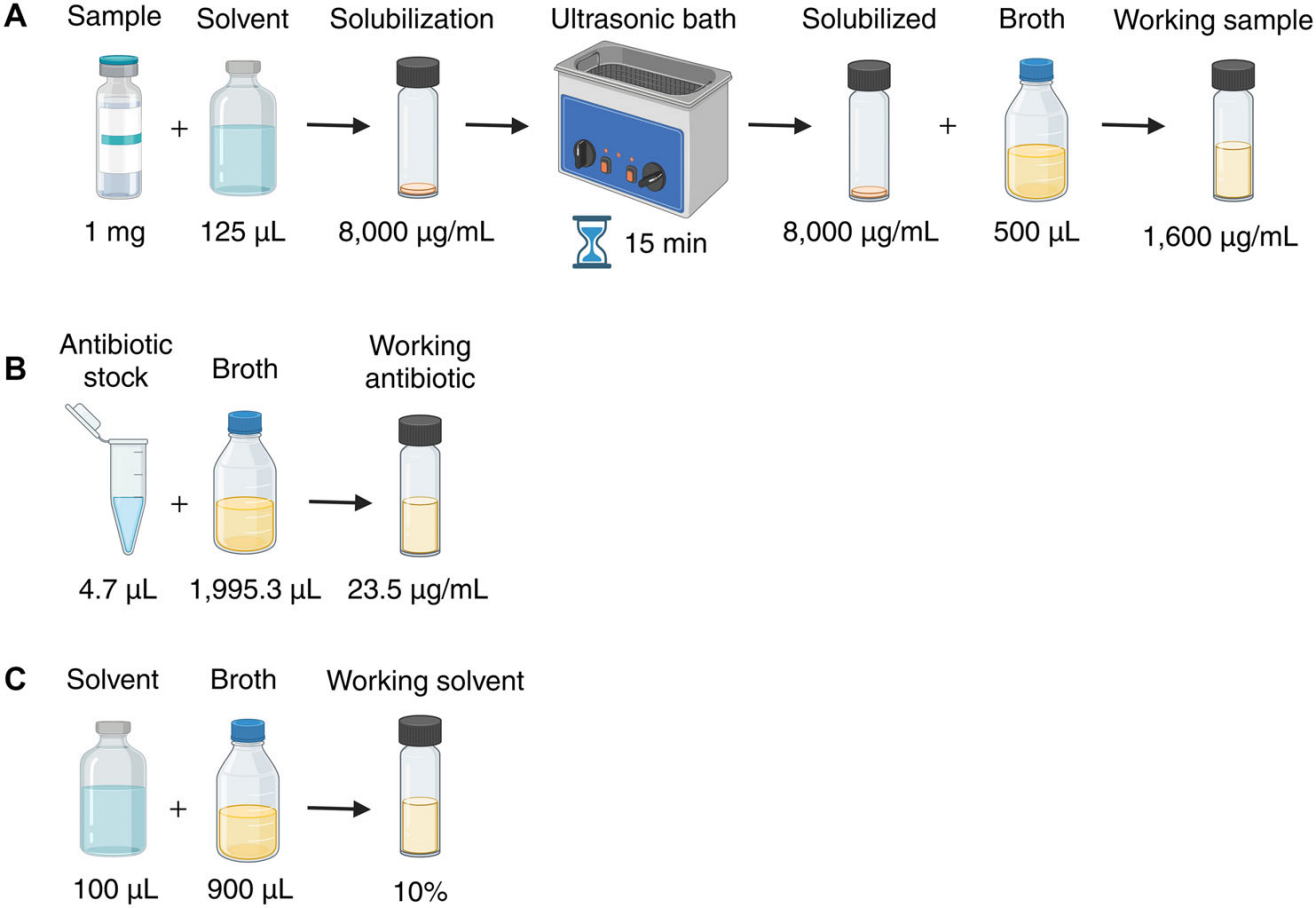
Representation of working solution preparation. **(A)** Steps to obtain the 4× concentrated working sample: Pure solvent is added to the sample for solubilization in an ultrasonic bath, and after solubilization, it is diluted in broth to achieve the desired concentration. In this protocol, the solution needs to be at a concentration of 1600 µg/mL to achieve a final well concentration of 400 µg/mL, where the solvent is at a concentration of 5% (v/v). **(B)** Steps to obtain the 4× concentrated working antibiotic: The antibiotic, previously prepared from a stock solution, is diluted in broth to achieve the desired concentration. In this protocol, the solution needs to be at a concentration of 23.5 µg/mL to achieve a final well concentration of 5.9 µg/mL. **(C)** Steps to obtain the 2× concentrated working solvent: The solvent is diluted in broth to obtain the desired concentration. In this protocol, the solution must be at a concentration of 10% (v/v) to achieve a final well concentration of 5% (v/v), which is the maximum evaluated concentration [Created in BioRender (UFU, L. (2026); https://BioRender.com/ug4wv4y].

The initial stage of the broth microdilution method involves preparing concentrated working solutions for the test sample, technical control (antibiotic), and solvent control. The current protocol is standardized for an initial sample concentration of 400 µg/mL and an initial antibiotic concentration of 5.9 µg/mL. Modifications can be made according to different assessment requirements; however, the working sample and working antibiotic solutions must always be prepared at 4× the highest desired final concentration. This process is presented as a workflow diagram in Figure 1.

### Materials

Test sample (natural product, isolated substance, or synthetic compound)
Dimethyl sulfoxide (DMSO) (Supelco, cat. no. 1.16743)
Supplemented Brucella broth (SBB) (see recipe)
Metronidazole stock solution (see recipe)

Flat‐bottom cylindrical test tubes, 5 mL
Analytical balance (Mettler Toledo, cat. no. 30697386)
Pipettes and tips, 20, 100, 200, and 1000 µL
Ultrasonic bath (Branson, cat. no. Z769614)

#### Prepare the working sample solution

1 Weigh 1 mg of the sample to be evaluated into a test tube using an analytical precision balance.
2 Dilute the sample by adding 125 µL of DMSO to the tube and homogenizing thoroughly.
  Other solvents may be used depending on specific requirements; however, it must always be evaluated whether the concentration used inhibits bacterial growth (see Basic Protocol 1, steps 9–10).
3 Place the sample in an ultrasonic bath for 15 min.
  This step ensures complete solubilization of the sample being evaluated.
4 Add 500 µL of SBB, resulting in a working sample solution concentration of 1600 µg/mL.
  While modifications are possible depending on assessment needs, the working sample solution must always be prepared at 4× the maximum desired final concentration.

#### Prepare the working antibiotic solution (technical control)

5 Remove the metronidazole stock solution (1000 µg/mL) from storage and allow it to thaw at room temperature.
6 After thawing, homogenize the stock solution thoroughly.
7 In a test tube, add 4.7 µL of the stock and 1995.3 µL of SBB.
8 Homogenize thoroughly to obtain the working metronidazole solution at a final concentration of 23.5 µg/mL.
  Metronidazole is used as a technical control against B. fragilis (CLSI, 2018), although other antibiotics and/or bacterial strains may be employed according to assessment needs; however, this solution must always be prepared at 4× the maximum desired final concentration for correct application in the protocol.

#### Prepare the working solvent solution (inhibition control)

9 In a test tube, add 100 µL of DMSO to 900 µL of SBB.
10 Homogenize thoroughly to obtain the working solvent solution at a concentration of 10% (v/v) DMSO.
  In this protocol, the final concentration of the solvent in the well is 5% (v/v). This same concentration must be evaluated to ensure that the solvent is not inhibiting bacterial growth, as many solvents can be toxic to bacterial cells (Ilieva et al., 2021; Martinez et al., 2008). Other solvents may be used, and the concentration may also be altered according to individual needs. Therefore, prepare the working solvent solution at 2× the final concentration used.
11 The prepared working solutions will be used in the setup of the 96‐well microplate (see Basic Protocol 2).

## Basic Protocol 2 PREPARATION OF THE 96‐WELL MICROPLATE

**Figure 2:**
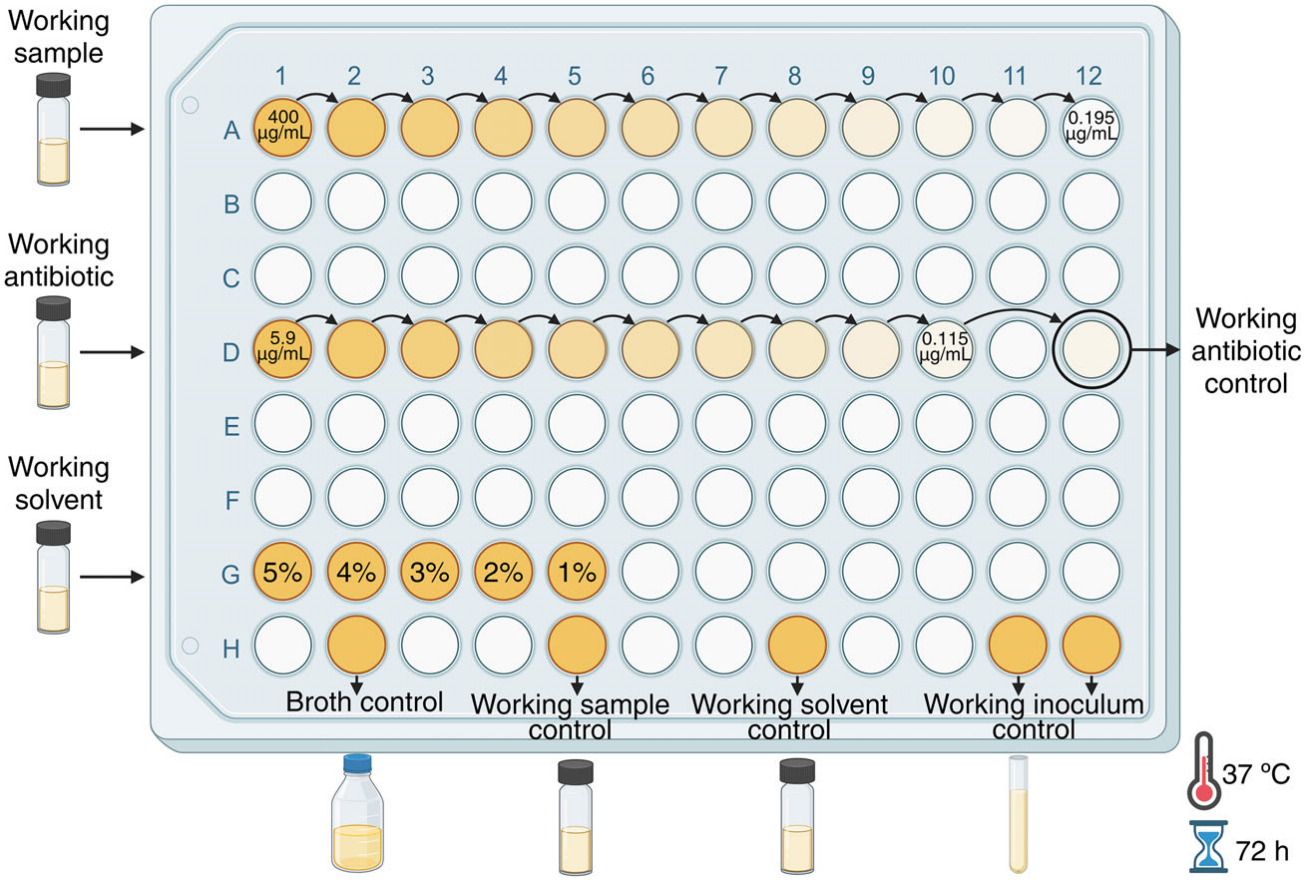
Schematic representation of the 96‐well microplate preparation. For the correct execution of the technique, the preparation of the 96‐well microplate must be carried out carefully, and the necessary controls must be included for validation. The sample evaluation is represented in row A, where broth is added to wells A1–A12; then the working sample is added to well A1 and serially diluted up to well A12, after which the same diluted volume is discarded from well A12 so that all wells have the same volume; in this way, a range of evaluated concentrations varying from 0.195 to 400 µg/mL is obtained. The working inoculum is added to all wells in the row. The technical control evaluation is represented in row D, where broth is added to wells D1–D10; the working antibiotic is added to well D1 and serially diluted up to well D10 and then discarded into well D12 so that all wells have the same volume, and well D12 serves as the antibiotic sterility control. The inoculum of the bacteria used as the technical control is added to wells D1–D10, resulting in the evaluated antimicrobial concentration in the technical control varying from 0.155 to 5.9 µg/mL. The solvent control is represented in row G, where the working inoculum of the bacteria evaluated in the sample and the working solvent are added to well G1, evaluating the highest concentration of the solvent used at 5% (v/v). When using a new unknown solvent, it is recommended that more solvent concentrations be evaluated (e.g., as represented in wells G2–G5). The sterility controls for the broth, working sample, and solvent are represented in wells H2, H5, and H8, respectively. No bacterial inoculum is added to these wells, and they should not present bacterial growth. Wells H11 and H12 are represented as bacterial growth controls, where only broth and the bacterial inoculum are added, and they must present growth. The addition of the bacterial inoculum to the microplate must strictly be carried out inside the anaerobic chamber to prevent the obligate anaerobic bacteria from contacting oxygen [Created in BioRender; UFU, L. (2026) https://BioRender.com/jpine52].

Basic Protocol 1 detailed how to correctly prepare the working solutions to be used for the antibacterial evaluation. Following this initial preparation, the next step consists of setting up the broth microdilution assay in a 96‐well plate. Depending on the number of samples to be tested, a single 96‐well plate may be completely filled, or more than one 96‐well plate may be required to perform the tests. This process is presented as a workflow diagram in Figure 2.

### Materials

Supplemented Brucella broth (SBB) (see recipe)
Working sample solution (see Basic Protocol 1, steps 1–4)
Working metronidazole solution (see Basic Protocol 1, steps 5–8)
Working solvent solution (see Basic Protocol 1, steps 9–10)

Biological safety cabinet (Veco, cat. no. EQ‐002/09‐C)
Anaerobic chamber (maintained at 10% CO_2_, 5%–10% H_2_, 85% N_2_ at 37°C; Don Whitley Scientific, cat. no. A03000)
Microplates, 96‐well (TPP, cat. no. Z707902)
Pipettes and tips, 20, 100, 200, and 1000 µL

1 Inside a biological safety cabinet, begin the preparation of the 96‐well microplate by adding 100 µL of SBB to all test wells (columns 1–12).
  Alternatively, this preparation can also be performed inside an anaerobic chamber, which would reduce the waiting time once the microplate is ready. However, space limitations inside the chamber often make it more practical to perform this step in a biological safety cabinet.
2 Add 100 µL of the 4× working sample solution to column 1 (e.g., well A1, B1, etc.).
3 To perform serial dilution, transfer 100 µL from column 1 to column 2. Mix thoroughly. Transfer 100 µL from column 2 to column 3. Repeat this process until column 12.
4 Discard 100 µL from column 12 so all wells contain 100 µL.
5 For the technical control, add 100 µL of SBB to all wells that will be tested (columns 1–10).
6 Add 100 µL of the 4× working metronidazole solution to column 1 (e.g., well A1, B1… etc.).
7 Repeat the process of serial dilution with the antibiotic for the technical control, but transfer 100 µL from column 10 to column 12, which will serve as a sterility control for the working antibiotic solution.
8 Prepare control wells:
  a. Bacterial growth control: 100 µL working solvent solution + 100 µL working inoculum (see Basic Protocol 3)
  b. Sterility: 200 µL SBB only
  c. Sterility: 150 µL SBB + 50 µL working sample solution
  d. Sterility: 100 µL SBB + 100 µL working solvent solution
  e. Bacterial growth control: 100 µL SBB + 100 µL working inoculum (see Basic Protocol 3)
9 Transfer the microplate immediately to the anaerobic chamber and incubate it for 2 h to allow gas exchange and oxygen reduction.
  If the preparation has already been performed inside the anaerobic chamber, this step can be omitted.
10 Following this period, the 96‐well microplate is ready for the addition of the bacterial inoculum (see Basic Protocol 3).

## Basic Protocol 3 INOCULUM STANDARDIZATION

**Figure 3:**
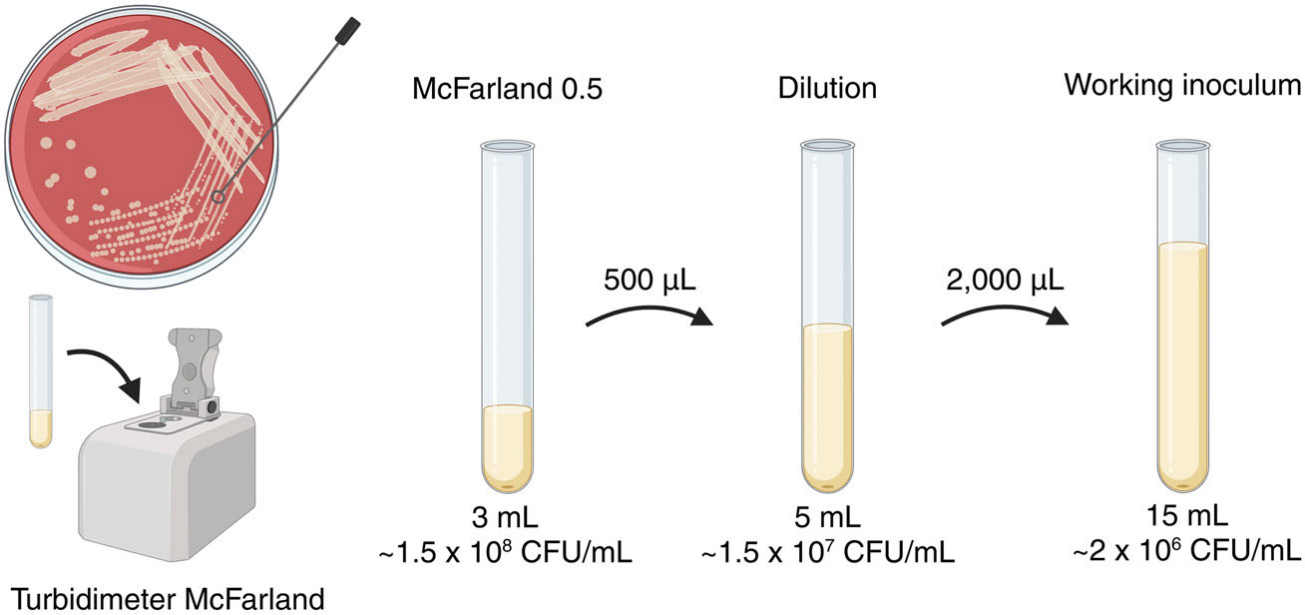
Workflow for inoculum standardization. The working inoculum is obtained through standardization using the McFarland scale as a reference, which indicates the approximate number of colony‐forming units (CFU) present based on turbidity. The working inoculum solution must be 2× concentrated. First, bacteria are grown on agar, and after the incubation period, fresh colonies are collected with a disposable loop and resuspended in broth, where a turbidimeter is used to reach the 0.5 McFarland scale, resulting in a concentration of ≈1.5 × 10^8^ CFU/mL. To reach the necessary concentration, it is recommended to perform two dilutions: the first at a 1:10 ratio and the second at a 1:7.5 ratio, in order to avoid dilutions involving the pipetting of small volumes, which can lead to technical errors. After performing the two dilutions, the working inoculum will be standardized at a concentration of ≈2 × 10^6^ CFU/mL, which is 2× concentrated compared to the required concentration of ≈1 × 10^6^ CFU/mL. This preparation is carried out completely inside the anaerobic chamber so that the obligate anaerobic bacteria do not come into contact with oxygen during any stage of the protocol [Created in BioRender; (UFU, L. (2026) https://BioRender.com/66ksvxj].

Following the setup of the 96‐well microplate described in Basic Protocol 2 using the working solutions from Basic Protocol 1, the next step of the experiment is the addition of the bacterial inoculum into the test wells. Given the oxygen sensitivity of anaerobic bacteria, strict adherence to the steps outlined in Basic Protocol 3 is essential for the correct execution of this protocol, and all bacterial manipulation must be performed inside the anaerobic chamber. This process is presented as a workflow diagram in Figure 3.

### Materials

Supplemented Brucella agar (SBA) (see recipe)
*B. fragilis* (ATCC 25285)
McFarland standard, 0.5 (DensiMAT densitometer ‐ BioMérieux cat. no. 99234).
Supplemented Brucella broth (SBB) (see recipe)
Anaerobic bacterial strains [e.g., *P. gingivalis* (ATCC 33277) and/or clinical isolates]

Disposable inoculating loop (Heathrow Scientific, cat no. HS81121D
Round‐bottom test tubes, 16 mL (Falcon, cat no. CLS352025)
Round‐bottom test tubes, 8 mL (Falcon, cat no. CLS352027)
Prepared 96‐well microplate (see Basic Protocol 2)
Anaerobic chamber (maintained at 10% CO_2_, 5%–10% H_2_, 85% N_2_ at 37°C; Don Whitley Scientific, cat. no. A03000)
Pipettes and tips, 20, 100, 200, and 1000 µL

1 Harvest colonies from a fresh (72 h) SBA culture of *P. gingivalis* and/or *B. fragilis* using a loop.
2 Resuspend in a test tube containing 3 mL of SBB to reach a turbidity equivalent to the 0.5 McFarland standard (≈1.5 × 10^8^ CFU/mL). Use a turbidimeter for precision.
3 Dilution 1 (1:10): Mix 500 µL of the 0.5 McFarland suspension with 4.5 mL of SBB.
4 Dilution 2 (1:7.5): Transfer 2000 µL of Dilution 1 into 13 mL of SBB. This is the standardized working inoculum solution (≈2 × 10^6^ CFU/mL).
5 Add 100 µL of the standardized working inoculum solution to each well containing the test compounds and controls (except sterility controls).
  The P. gingivalis inoculum must be added to the wells used to analyze the sample, solvent (bacterial growth control), and inoculum (bacterial growth control). The B. fragilis inoculum must be added to the wells that evaluate the technical control (metronidazole) and inoculum (bacterial growth control).
6 Each well now contains a volume of 200 µL, the compound is at 1× concentration, and the bacterial density is ≈1 × 10^6^ CFU/mL. The total concentration of the solvent (DMSO) utilized is 5% (v/v), which is the same concentration that must be assessed to verify that the solvent does not inhibit the bacterial cells.
7 Incubate the microplates in the anaerobic chamber at 37°C for 72 h. Following this period, read and interpret the results (see Basic Protocol 4).

## Basic Protocol 4 READING AND INTERPRETING THE RESULTS

**Figure 4:**
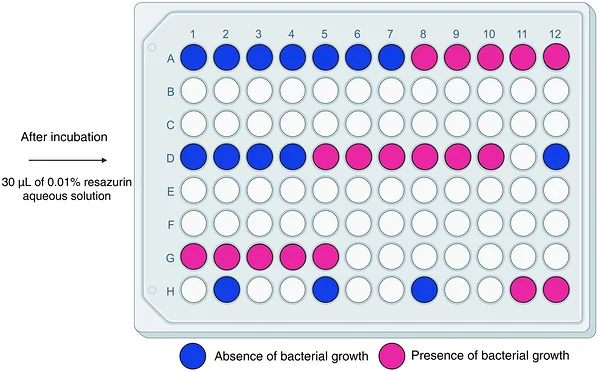
Illustration of result interpretation. After the microplate incubation period, a 0.001% (w/v) resazurin aqueous solution is used to obtain the results; the presence of bacterial growth results in oxido‐reduction, and the blue‐colored resazurin is converted to pink‐colored resofurin. The MIC result is defined by the lowest evaluated concentration that does not present bacterial growth. Thus, as represented in **Figure** **3**, the MIC of the sample is determined in well A7, which corresponds to the concentration of 6.25 µg/mL, and the MIC of the technical control is determined in well D4, corresponding to the concentration of 0.7375 µg/mL. The solvent did not inhibit bacterial growth at the concentrations evaluated, as wells G1–G5 presented bacterial growth. The sterility controls for the broth (H2), sample (H5), and solvent (H8) did not present bacterial growth. The inoculum controls in H11 and H12 presented bacterial growth. The addition of resazurin must occur inside the anaerobic chamber because contact with oxygen can kill the obligate anaerobic bacteria, leading to incorrect results; however, the reading must be done within a maximum of 20 min so that the anoxic and/or acidic environment does not cause nonspecific reduction of the resazurin, which can also lead to incorrect results and the need for experiment repetition [Created in BioRender; UFU, L. (2026) https://BioRender.com/03vo9s0].

Following the inoculum addition and incubation period of the 96‐well microplate described in Basic Protocol 3, the next step of the experiment involves reading and interpreting the results, which are detailed in Basic Protocol 4. In this protocol, resazurin is used as a developer; the methodology previously standardized by Sarker et al. (2007) was adapted to allow the determination of the MIC based on the color change of resazurin. A blue color indicates the absence of bacterial growth, whereas a pink color indicates the presence of bacterial growth. This process is presented as a workflow diagram in Figure 4.

### Materials

Resazurin, 0.01% (see recipe; Sigma–Aldrich, cat no. R7017‐5G)

96‐well microplate after incubation (see Basic Protocol 3)
Anaerobic chamber (maintained at 10% CO_2_, 5%–10% H_2_, 85% N_2_ at 37°C; Don Whitley Scientific, cat. no. A03000)
Pipettes and tips, 20, 100, 200, and 1000 µL

1 Add 30 µL of 0.01% (w/v) resazurin aqueous solution to each well (inside the anaerobic chamber).
2 Incubate the plates again in the anaerobic chamber and wait ≈20 min to allow color development.
  Do not exceed 20 min. Prolonged exposure of resazurin in an anoxic environment and/or under acidic conditions can cause “over‐reduction” to dihydroresorufin (colorless or yellowish), leading to false results.
3 Visually assess the color change.

## REAGENTS AND SOLUTIONS

*Use deionized, distilled water in all recipes and protocol steps*.

### Hemin solution (5000 µg/mL)

Weigh 0.05 g of hemin (Sigma–Aldrich, cat. no. H9039).
Add 1 mL of 1 M NaOH (see recipe).
Add 9 mL of distilled water.
Mix well and filter using a syringe filter (0.22‐µm pore size; Kasvi, cat. no. K18‐2522PES‐E)
Store at 4°C in a light‐protected container for a maximum of 30 days.

### Menadione solution (1000 µg/mL)

Weigh 0.01 g of menadione (Sigma–Aldrich, cat. no. M5628).
Add 10 mL of absolute ethanol.
Mix well and filter using a syringe filter (0.22‐µm pore size; Kasvi, cat. no. K18‐2522PES‐E).
Store at 4°C in a light‐protected container for a maximum of 30 days.

### Metronidazole stock solution (1000 µg/mL)

Weigh 10 mg of metronidazole (Sigma–Aldrich, cat. no. M3761).
Add 10 mL of sterilized water.
Mix well.
Aliquot and store indefinitely at –80°C.

### NaOH solution (1 M)

Weigh 1.0 g of sodium hydroxide (Sigma–Aldrich, cat. no. 221465).
Add 25 mL of distilled water.
Mix thoroughly.
Filter the solution (e.g., syringe filter; 0.22‐µm pore size; Kasvi, cat. no. K18‐2522PES‐E).
Store at room temperature for up to 6 months.

### Resazurin solution, 0.01% (w/v)

Weigh 2 mg of resazurin (Sigma–Aldrich, cat. no. R7017).
Add 20 mL sterile distilled water.
Mix well.
Store protected from light; it can be stored under refrigeration at 4°C for 7 days.

### Supplemented Brucella agar (SBA)

Weigh 21.5 g of Brucella agar (Gibco, cat. no. 211086).
Add 500 mL of distilled water.
Sterilize by autoclaving at 121°C for 15 min.
Cool to 60°C.
Add 500 µL of menadione solution (see recipe).
Add 500 µL of hemin solution (see recipe).
Add 25 mL of defibrinated horse blood.
Pour the medium into 100 mm Petri dishes (Sigma‐Aldrich, cat. no. P5731)
Store under refrigeration at 4°C for 30 days.
Prior to use, place the medium inside the anaerobic chamber for 24 h to allow gas exchange and oxygen reduction.

### Supplemented Brucella broth (SBB)

Weigh 7 g of Brucella broth (Gibco, cat. no. 211088).
Add 250 mL of distilled water.
Sterilize by autoclaving at 121°C for 15 min.
Cool to 60°C.
Add 250 µL of menadione solution (see recipe).
Add 250 µL of hemin solution (see recipe).
Store under refrigeration at 4°C for 30 days.
Prior to use, place the medium inside the anaerobic chamber for 24 h to allow gas exchange and oxygen reduction.

## COMMENTARY

### Critical Parameters

Certain aspects, such as appropriate culture medium and supplementation, antibiotics, and incubation conditions (time and temperature), for each bacterial species must be considered when adapting the protocol. It is crucial to know the type of sample that will be used as an antibacterial agent to employ the appropriate solvent for each sample and ensure its stability under anaerobic conditions.

The initial concentration of the working sample solution can also be adapted to better suit the work. In the literature, there are different criteria for evaluating the activity of natural extracts, isolated substances, and synthetic compounds. Our study group mainly uses two of them. If the extract presents a MIC value less than 100 µg/mL, it is considered to have good antibacterial activity; from 100 to 500 µg/mL, it is considered moderate; 500 to 1000 µg/mL indicates weak antibacterial activity; and over 1000 µg/mL, it is considered inactive (Holetz et al., 2002). For isolated compounds, the presence of activity is very interesting in the case of concentrations below 10 µg/mL (Rios & Recio, 2005).

Constant attention must be paid to the final concentration of the working solvent solution, as well as to sterility controls of the broth culture medium, working sample, solvent, and antibiotic solutions. They are essential to guarantee that the bacterial growth observed is due exclusively to the working inoculum solution added to the well and not to external contamination, and that the absence of bacterial growth observed is due exclusively to the activity of the antimicrobial agent tested and not to the solvent concentration used. The working inoculum solution control is also important to confirm bacterial growth during the experiment. All controls ensure the validity and reliability of the MIC results.

It is recommended that tests be performed in triplicate for statistically meaningful results. Prior technical training and an anaerobic chamber are needed to implement this protocol.

### Troubleshooting Table

Table 1 presents the potential problems and solutions.

**Table 1:** Troubleshooting Table

Problem	Possible cause	Solution
No bacterial growth with working solvent solution	Solvent inhibiting bacterial growth	Evaluate different concentrations of the solvent to find a safe concentration for use.
		Use a different type of solvent that is not toxic to bacterial cells.
Precipitation in wells	Compound insolubility	Check solvent compatibility.
No growth on SBA	Oxygen exposure during setup	As these are obligate anaerobes, minimal exposure to oxygen can result in bacterial death. Always ensure that manipulation is performed inside the anaerobic chamber and using culture media in which the oxygen concentration has been reduced.
No growth in positive control	Oxygen exposure during setup	Ensure SBB was pre‐reduced for 24 h.
	Inoculum too low	Use a calibrated turbidimeter. Verify colony viability (fresh 72 h culture).
All wells turn pink	Contamination	Check sterility controls. Always maintain proper microbiological handling techniques.
	Reduction due to lack of oxygen	Do not leave resazurin plates in the anoxic environment for prolonged periods (max. 20 min).
Wells turn colorless after pink	Over‐reduction	Read results immediately (within 20 min).
Technical control outside the range	Error in performing the serial dilution	Verify the calibration of the pipettes and ensure technical training for optimal pipetting.
	Problems with the antimicrobial	Use antimicrobial agents from reliable brands and within their expiration date.
		Verify the calculations and, if necessary, re‐prepare a new batch of the stock solution.
Nonlinear result	Error in performing the serial dilution	Verify the calibration of the pipettes and ensure technical training for optimal pipetting.

### Understanding Results

This protocol provides a robust and reproducible method for determining the MIC of compounds against obligate anaerobic bacteria, such as *P. gingivalis*. The key to success is the rigorous maintenance of anaerobic conditions and the accuracy of the inoculation. The MIC is defined as the lowest concentration of the tested compound in the microplate that results in visible inhibition of bacterial growth (Andrews, 2001). The oxidation–reduction indicator resazurin is blue in its oxidized state and turns pink (resorufin) in its reduced state, indicating metabolic activity and, consequently, bacterial growth (Sarker et al., 2007). Inhibition is determined by the absence of color change (i.e., the well remains blue) after incubation with resazurin. In a serial dilution (Columns 1–12), a clear transition is expected to be observed, where wells with the highest concentration of the antibacterial agent tested or antibiotic (e.g., Column 1) remain blue, and wells with the lowest concentration (e.g., Column 12) turn pink (growth).

**Figure 5:**
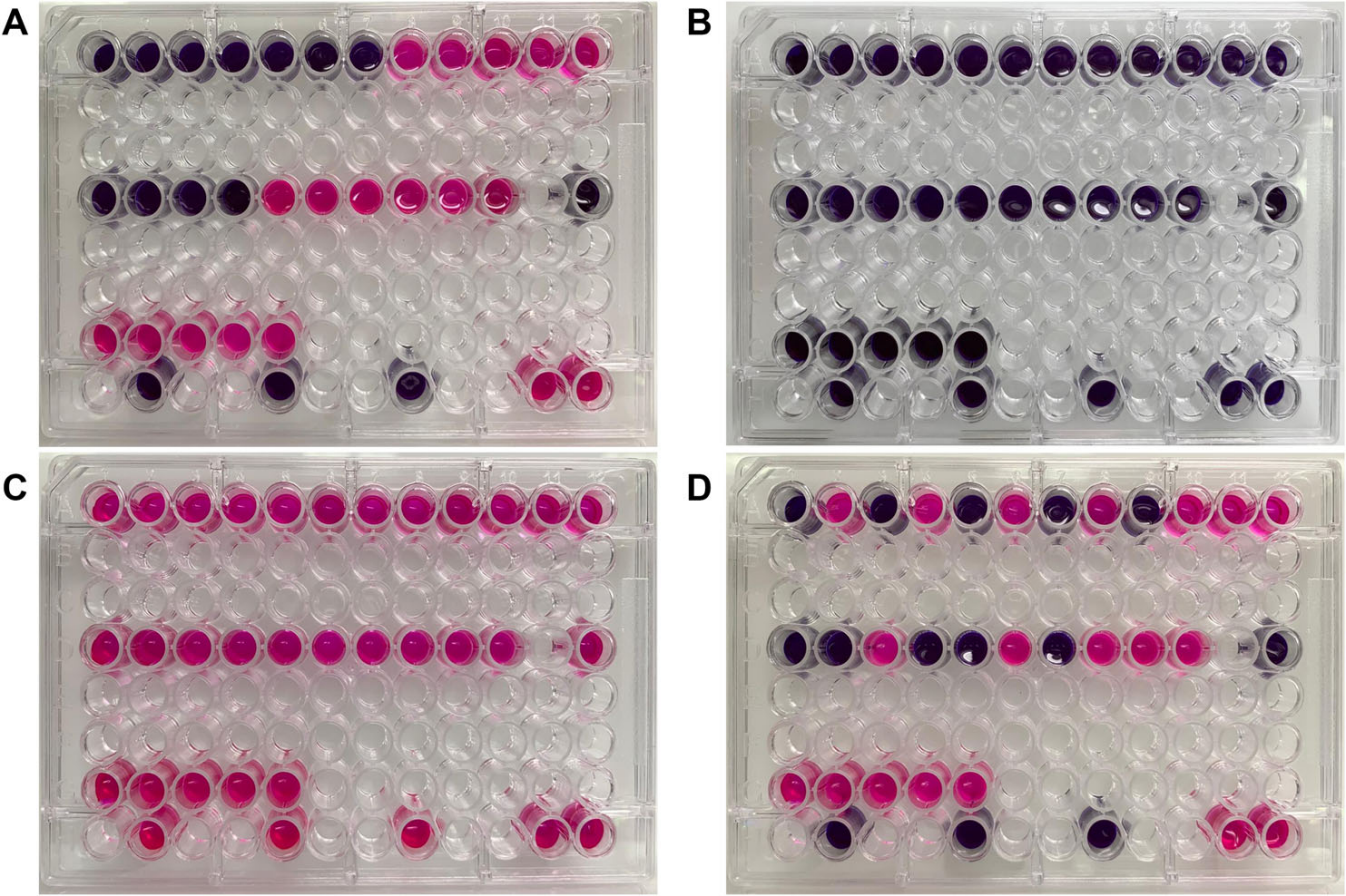
Possible MIC results obtained after the addition of resazurin. **(A)** Ideal result to be observed: The MIC of the sample against *P. gingivalis* was determined in well A7 (6.25 µg/mL), the technical control was validated with the metronidazole MIC against *B. fragilis* (ATCC 25285) in well D4 (0.7375 µg/mL, within the recommended range), and all sterility controls (metronidazole D12, broth H2, sample H5, and solvent H8) showed no color change; furthermore, the solvent control wells (G1–G5) showed no inhibition, and the growth controls for *P. gingivalis* (H11) and *B. fragilis* (H12) turned pink, indicating bacterial growth. **(B)** Result when MIC determination is not possible due to the absence of bacterial growth: The indication is that the bacterial growth controls for *P. gingivalis* (H11) and *B. fragilis* (H12) remain blue. **(C)** Result when MIC determination is not possible due to contamination and/or "over‐reduction" caused by lack of oxygen or acid‐producing bacterial strains: This is indicated by the sterility controls (metronidazole D12, broth H2, sample H5, and solvent H8) showing a color change. **(D)** Result when MIC determination is not possible due to nonlinear serial dilution: Even with valid sterility controls (blue) and growth controls (pink), the MIC determination for the sample (row A) and metronidazole (row D) is impossible because the color varied nonlinearly between blue and pink. (Created by the authors)

In Figure 5, a combination of possible assay results can be observed. The ideal result occurs when it is possible to determine the MIC of the evaluated sample for *P. gingivalis*, in addition to the working solvent and the working inoculum control exhibiting bacterial growth, and all sterility controls showing no bacterial growth (Figure 5a). Row A exemplifies a sample with a MIC result of 6.25 µg/mL. Row D exemplifies the antibiotic with a MIC result of 0.7375 µg/mL. The technique used is validated when the MIC of metronidazole is within the range determined by CLSI (*B. fragilis* ATCC 25285: metronidazole MIC range 0.25–2 µg/mL) (CLSI, 2018).

Unsatisfactory results occur when all wells (especially the inoculum growth control well) remain blue (Figure 5b), indicating that no bacterial growth occurred, possibly due to oxygen exposure of the strain. Another unsatisfactory result is when all wells of the microplate turn pink (Figure 5c) or *colorless/yellowish*, indicating that the absence of oxygen for a prolonged period of time or acid‐producing bacterial strains caused the non‐specific oxidation–reduction (Njoku et al., 2023) of resazurin or indicating external contamination of any of the materials used, reinforcing sterility precautions during the experiment. Finally, when the result is nonlinear, where the coloration reverses between pink and blue (Figure 5d), this may indicate a technical error in performing the serial dilution, making it impossible to determine the MIC and indicating the need for technician training.

### Time Considerations

Timing information can be found in Table 2. The timeframes for performing the steps were calculated based on teams that have received training and are proficient with the technique; variations may occur depending on the level of technical expertise.

**Table 2:** Time Required for Protocol Steps

Days	Steps	Time
Day 1	Inoculating the bacteria onto SBA	72 h
Day 4	Basic protocol 1 steps 1–11	≈1–2 h
	Basic protocol 2 steps 1–10	≈1–3 h
	Basic protocol 3 steps 1–6	≈45 min
	Basic protocol 3 step 7	72 h
Day 6	Basic protocol 4 steps 1–3	≈30 min

### Author Contributions

**Daniela Silva Gonçalves**: Conceptualization; visualization; writing—original draft; writing—review and editing. **Mariana Brentini Santiago**: Conceptualization; visualization; writing—original draft; writing—review and editing. **Lucas Andrade Sousa**: Conceptualization; writing—review and editing. **Carlos Henrique Gomes Martins**: Conceptualization; funding acquisition; supervision; writing—review and editing.

### Conflict of Interest

The authors declare no conflict of interest.

## Data Availability

Data sharing is not applicable to this article as no new data were created or analyzed in this study.
